# Epithelial and mesenchymal progenitor cells in normal and inflamed human lacrimal glands

**DOI:** 10.1016/j.exer.2025.110590

**Published:** 2025-08-21

**Authors:** Mohammad Gufran Siddiqui, Tejaswini Pingali, Saumya Jakati, Vivek Singh, Sayan Basu, Swati Singh

**Affiliations:** a Center for Ocular Regeneration (CORE), Prof. Brien Holden Eye Research Centre (BHERC), L V Prasad Eye Institute, Hyderabad, India; b Manipal Academy of Higher Education (MAHE), Manipal, India; c Hariram Motumal Nasta and Renu Hariram Nasta Ophthalmic Plastic Surgery Services, L V Prasad Eye Institute, Hyderabad, Telangana, India; d Ophthalmic Pathology Laboratory, LV Prasad Eye Institute, Hyderabad, Telangana, India; e Shantilal Shanghvi Cornea Institute, L V Prasad Eye Institute, Hyderabad, Telangana, India; f Prof. Krothapalli Ravindranath Ophthalmic Research Biorepository, LV Prasad Eye Institute, Hyderabad, India

**Keywords:** Lacrimal gland, Regeneration, Stem cells, Progenitor cells, Nestin, Dacryoadenitis

## Abstract

Stem/progenitor cells play an important role in tissue repair and regeneration in response to injury and maintain tissue homeostasis. The existence of the progenitor cells within the human lacrimal gland is established, but their distribution and response to tissue injury are unclear. This study investigated progenitor cells’ distribution and gene expression in normal and inflamed human lacrimal glands. Biopsies from healthy human lacrimal glands (n = 9, 61 ± 14.3 years) and non-specific dacryoadenitis (n = 5; 42.8 ± 19.3 years) were immunostained with progenitor cell markers- Nestin, p63 alpha, CK15, ABCG2, c-kit, and CD90, along with RT-PCR. The basal cell layer of interlobular and intercalated ducts and periacinar spindle-shaped cells expressed progenitor markers. In the dacryoadenitis specimens, lacrimal gland injury was noted as moderate to severe diffuse inflammation and acinar atrophy. The average percentage of positive cells (in ten high-power fields) showed no significant change in dacryoadenitis specimens, except for an increase in CD90. Gene expression revealed a substantial increase in CD90 and reduced expression of ABCG2 and p63α in dacryoadenitis. Double immunostaining with CD73 and CD105 demonstrated predominant CD90 expression in the endothelial cells rather than in the periacinar or periductal regions. The human lacrimal gland has progenitor cells around the intercalated and interlobular ducts that do not increase in severe glandular inflammation. Inflamed lacrimal glands have a demonstrable increase in the expression of MSCs but a reduction in epithelial progenitor cells. Future studies on the interaction between immune and progenitor/stem cells within the lacrimal gland will clarify the mechanisms involved.

## 1 Introduction

The lacrimal gland produces an aqueous component of tears, and its involvement with inflammation results in aqueous-deficient dry eye (ADDE) disease and ocular surface morbidity (Singh and Basu, 2020). The majority of ADDE patients develop symptomatology late in the disease when the gland is atrophied (Singh et al., 2021). The most common cause of ADDE is Sjögren’s syndrome (SS), which is an autoimmune disease characterized by salivary and lacrimal gland inflammation, acinar destruction, and eventual atrophy. As no curative therapy can re-establish the tear secretion from the lacrimal gland, current research is focused on regenerating the lacrimal gland in vitro or in vivo via injecting mesenchymal stem cells (MSC) in animal models to reverse the glandular inflammation (Joshi et al., 2023). Tissue-resident stem cells in the lacrimal glands have been studied for over 20 years. The presence of stem/progenitor cells within mouse and human lacrimal glands has been demonstrated in culture and immunofluorescence studies (Ackermann et al., 2015; Gromova et al., 2017; Hawley et al., 2017; Hirayama and Hiraya-Ma, 2018; Lin et al., 2017; Liu et al., 2017; Shatos et al., 2012; Tiwari et al., 2012; You et al., 2011; You et al., 2011; Zoukhri et al., 2008). Lineage tracing studies in the mouse lacrimal glands have proposed the progenitor cells to be acinar, ductal, and myoepithelial. Still, it is unclear where the stem cells are in the human lacrimal glands (You et al., 2011). Though no consensus exists on the stem cell marker specific to the lacrimal glands, various studies have proposed different markers, mainly c-kit, Sox2, Nanog, nestin, FGF10, and Oct4 (Ackermann et al., 2015; Tiwari et al., 2012). These markers were studied in lacrimal gland cultures. There is a lack of clarity on the luminal or basal localization of these cells in the lacrimal glands.

Also, the behavior of the lacrimal gland stem/progenitor cells in response to gland inflammation, like in ADDE, is unknown. There are no studies on the lacrimal glands of SS patients, as lacrimal gland biopsy is not routinely performed in SS patients. It is essential to learn the interactions between the lacrimal gland progenitor cells and immune cells in glandular inflammation, whether they proliferate in inflamed glands or co-localize to areas with acinar atrophy or inflammation. Recent research is exploring the MSCs injection to repair the lacrimal glands of ADDE patients, and it is crucial to understand what happens to the native epithelial or mesenchymal stem cells of the lacrimal gland in the inflammatory milieu (Singh and Selva, 2022). Dacryoadenitis denotes inflammation of the lacrimal gland of non-specific or specific etiologies (Singh et al., 2023). A biopsy is performed in dacryoadenitis cases for pathological tissue diagnosis. The tissue biopsy from inflamed glands can help understand the relationship/response of glandular acinar tissue/stem cells to glandular inflammation (Singh et al., 2023). We hypothesize that the lacrimal gland stem cell population would increase in inflamed glands to repair the glandular cells. Hence, the current study evaluated the distribution of stem/progenitor cell markers in healthy human lacrimal glands and glands affected with inflammation, i.e., dacryoadenitis. A panel of potential progenitor cell markers was selected based on published studies, and gene expression was carried out for the markers.

## 2 Methods

### 2.1 Normal human lacrimal gland tissue

**Fig. 1:**
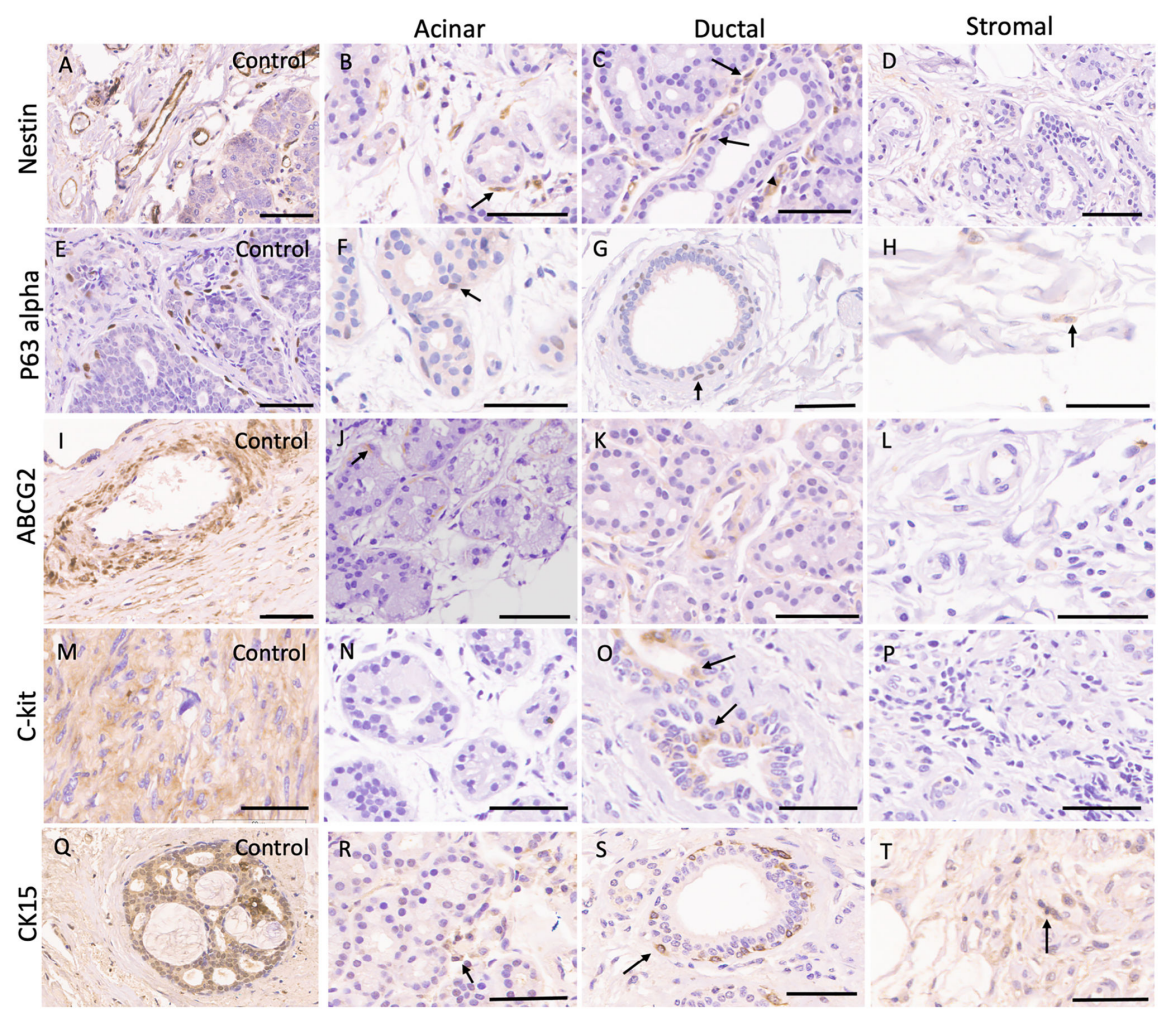
Stem/progenitor cells in normal human lacrimal gland (each scale bar represents 50 pm). A-D, Nestin expression in vessels of normal lacrimal gland tissue (positive control, A), myoepithelial cells (B, marked with an arrow), vessels around interlobular ducts (C, marked with an arrow), and negative expression in the stroma (D). E-H, P63 alpha expression in normal lacrimal gland (positive control, E), basal cells of intralobular (F, marked with an arrow) and interlobular ducts G, marked with an arrow), and scattered focal positivity in the stroma (H). I-L, ABCG2 expression in placental blood vessels (positive control, I), periacinar myoepithelial cells (J, marked with an arrow), and negative expression in ducts and stroma (K, L). M-P, c-kit positivity in gastrointestinal tumor (positive control), no expression in acini (N), positive staining in ductular epithelium (O, marked with an arrow), and no expression in stroma (P). Q-T, CK15 expression in cells of mucinous adenocarcinoma of breast (positive control, Q), intralobular ducts (R), basal cells of interlobular ducts (S, marked with an arrow), and stromal cells (O).The control tissues are salivary glands (Nestin), squamous cell carcinoma (P63alpha), placental tissue (ABCG2), gastrointestinal stromal tumor (c-kit), and mucinous carcinoma of the eyelid (CK15), respectively.

The whole orbital lobe of the lacrimal gland obtained from fresh body donors (n = 9; five males, mean age 61 ± 14.3 years) and lacrimal gland biopsies from patients with dacryoadenitis (n = 5; two males, mean age 42.8 ± 19.3 years) were examined using routine histology, immunohistochemistry, and gene expression studies. The study complied with the declarations of Helsinki, and the institutional ethics committee (LV Prasad Eye Institute Ethics Committee; LEC-BHR-01-20-378) approved the study. A trained oculoplastic surgeon (S.S) obtained the orbital lobe of the lacrimal gland from the eyebank donation calls (within 90 min of death) after the eye bank staff procured the donor corneas (Fig. 1). Written informed consent was obtained from the relatives before the sample collection. The donors’ medical records and ocular histories revealed no known lacrimal gland or ocular surface pathology.

### 2.2 Glands affected with dacryoadenitis

Paraffin-embedded and fresh frozen tissue biopsies of chronic non-specific dacryoadenitis (n = 5; two males, mean age 42.8 ± 19.3 years) were used for immunostaining. The specimens were selected from the tissue biopsies available in biobank at -80°. Specific dacryoadenitis, i.e., sarcoidosis, IgG4-related dacryoadenitis, Wegener’s granulomatosis, and microscopic polyangiitis were ruled out based on clinical examination, radiology, and serological analysis (Singh and Selva, 2022). Only non-specific dacryoadenitis cases with mixed inflammatory mononuclear cell populations and not on oral steroids were analyzed. The average disease duration was 5.7 ± 4.4 months. The only clinical complaint was upper eyelid swelling with ptosis in all patients. None of the patients had dry eye disease (based on Schirmer 14 ± 3.1, no corneal fluorescein staining). These patients had negative systemic workups (antinuclear antibody profile, serum ACE, normal chest X-ray). The histological changes seen on Hematoxylin-Eosin staining were defined in terms of acinar atrophy (mild, moderate, severe), and degree of inflammation (<30 cells/HPF), moderate (30–100 cells/HPF), severe (>100 cells/HPF).

### 2.3 Immunohistochemistry

According to an earlier published protocol, normal and dacryoadenitis lacrimal gland biopsies were stained for immunohistochemical antibody markers (Singh et al., 2023). The antibodies used were - mouse monoclonal anti-Nestin (sc-23927, 1:100, Santa Cruz biotechnologies, Texas, USA), mouse monoclonal anti-c-kit (sc-393910, 1:100, Santa Cruz biotechnologies, Texas, USA), mouse monoclonal anti-cytokeratin 15 (CK15) (sc-47697, 1:100, Santa Cruz biotechnologies, Texas, USA), mouse monoclonal anti-ABCG2 (sc-377176, 1:100, Santa Cruz biotechnologies, Texas, USA), sheep polyclonal CD90 (af2067, 1:100, R&D systems, USA) and mouse monoclonal anti-p63α (sc-5301, 1:100, Santa Cruz biotechnologies, Texas, USA). Briefly, deparaffinized sections were rehydrated with graded alcohol, followed by antigen retrieval and slides incubation with bovine serum albumin (catalog no A7906, Sigma, USA) to avoid non-specific antibody reaction. The primary antibodies were applied for 1 h at room temperature. The prediluted secondary antibodies (Dako, Denmark, Europe) were incubated at room temperature for 30 min. Three slides were analyzed under an Olympus light microscope (CX21i), and images were captured using Aperio image scope software (Leica Biosystems, Mumbai, India). The slide with the maximum number of positively staining cells was used for the IHC profiler (quantitative analysis) and qualitative description. The stained images were analyzed using the IHC profiler plugin in Image J for quantitative analysis (S.S., S.J.). IHC profiler is an automated algorithm-based digital image analyzer that quantifies the antibody staining intensity in IHC sections (Varghese et al., 2014). Ten 40X fields of each slide were evaluated, and cells showing IHC expression were reported as percentages (Varghese et al., 2014). Only the positive and highly positive % cell counts of the IHC profiler were taken for calculations. A low positive cell count was excluded. The % positive cell count was calculated using an IHC profiler (excluding the inflammatory cells) for dacryoadenitis biopsies in the acini-containing areas. For qualitative description, based on the deposition of the Diaminobenzidine Chromagen, the staining was interpreted as a type of cell stained (peri-acinar, peri-ductal, stromal), location within the cell (nuclear, cytoplasmic, or membranous), and the intensity (1+, 2+,3+) of the staining.

### 2.4 Double immunostaining

For MSCs (as they are involved in tissue repair), double immunostaining was performed with three MSC markers to confirm their presence and location in both normal and inflamed glands. 10 % neutral-buffered formalin-fixed lacrimal gland tissues were processed using an automated tissue processor (Leica, TP1020) followed by a microtome (Leica, RM2125RTS) to obtain 4-5 μm-thick paraffin-embedded tissue sections. Paraffin crosslinked tissues were deparaffinized on a hot plate set at 70 °C for 20 min, followed by three xylene washes for 5 min each. The tissues were rehydrated using alcohol washes (100 %, 90 %, 80 %) for 5 min each. The tissues were next subjected to antigen retrieval using citrate buffer (pH 6.0), followed by three 1X Phosphate buffer saline (PBS, pH 7.0) washes for 5 min each. The PBS was filtered with a PES 0.22 Sterile filter (GVS Filtrations USA). To permeabilize the sample for cytoplasmic and nuclear markers, the tissues were incubated with 0.5 % Triton X, 20 min at room temperature (RT), followed by three PBS washes, 10 min each. The tissues were then blocked using 2.5 % bovine serum albumin (BSA) (A7906, Sigma-Aldrich, St. Louis, USA) for 45 min at RT. The primary antibodies CD73 (rabbit,13160S; 1:150), and CD105 (mouse, sc376381; 1:150), and CD90 (mouse, sc59396; 1:150) were used individually and in combinations: CD90^+^CD73 and CD105 + CD73 and incubated overnight at 4 °C. Negative controls were incubated with BSA alone. The following day, tissue sections were incubated with secondary antibodies (A11012; anti-rabbit 594 and A11001; anti-mouse 488) for 45 min at RT. The 4, 6-diamidino-2-phenylindole (ab104139, Abcam, Cambridge, UK) was used for counterstaining the nucleus, followed by mounting the slides and capturing images using a fluorescent microscope (Zeiss, Scope A1, Germany).

### 2.5 Gene expression analysis

The frozen lacrimal gland tissue samples (control 4; two males, 54.6 ± 13.6 years) and inflamed 5 (three males, 31 ± 10.8 years) were chopped into fine pieces. Three of the five inflamed biopsies were from the same set of tissues as used for immunostaining, but two were taken afresh. Total RNA isolation was carried out using a phenol-chloroform-based Trizol method. The spectrophotometric reading at a 260/280 wavelength ratio stated the quality and quantity of RNA and quantified it. Following the manufacturer’s protocol, using the superscript IV kit method, one μg of RNA was used to synthesize complementary DNA from control and inflamed tissues (Invitrogen, 18090050 Lithuania, Europe). This was followed by gene expression studies for the following genes- CD90, CK15, Nestin, ABCG2, p63α, and β-actin (housekeeping gene) through quantitative real-time polymerase chain reaction (qRT-PCR) using the DyNAmo Color Flash SYBR Green qPCR Kit (F-416L, Thermo Fisher Scientific, USA) and Bio-Rad CFX OPUS 96 RealTime PCR (Bio-Rad Laboratories, USA). Thermal cycling conditions included initial enzyme activation at 95 °C for 10 min, followed by 33 cycles of denaturation at 95 °C for 30 s, annealing at 55 °C/60 °C for 30 s, and elongation at 72 °C for 30 s. The details of the primer sequences and the size of the amplicon for each of the genes have been mentioned in Table 3. Gene expression was normalized to the housekeeping gene β-actin, and the relative fold change was calculated using the 2^(-ΔΔCT) method. We did not check for c-kit as the primer standardization did not work.

### 2.6 Statistical analysis

The IHC positivity for progenitor markers was compared using the Mann-Whitney test (GraphPad Prism 10 software, USA). Mean fluorescence intensity was calculated using ImageJ-win64 software. The graphs were plotted using the mean grey value of each marker against each sample (control v/s inflamed; unpaired *t*-test at p < 0.05).

Quantitative real-time PCR results were calculated using MS Excel version 10. The graphs were plotted using the Mann-Whitney test in GraphPad Prism (GraphPad Software, San Diego, USA, version 10.0; p < 0.05) using relative fold change values against each gene specified.

## 3 Results

### 3.1 Normal human lacrimal gland

The expression of markers was not uniform across the specimens (Fig. 1). The progenitor cells in adult human lacrimal glands constituted less than 1 % of the cells seen in a 40X field (Table 1). The expression was nuclear for p63α and cytoplasmic for CK15, ABCG2, Nestin, and c-kit (Table 2). The location was periductal for p63α, CK15, and periacinar for ABCG2 (Fig. 1). Nestin-positive cells were located around the acini precisely like the myoepithelial cells in morphology and location (Fig. 1B). Also, intercalated, and interlobular ducts expressed Nestin positive cells in the basal layer (Fig. 1C). ABCG2 positive cells were spindle-shaped cells surrounding the acini, matching the location of myoepithelial cells (Fig. 1J–L). No ABCG2 positivity was seen in intercalated or interlobular ducts. c-kit positivity was observed in ductular epithelium (Fig. 1N–P). In most samples, the cytoplasmic CK15 staining was positive in the stromal cells (having round nuclei) surrounding the acinar area. Also, CK15 positivity in endothelial and basal epithelial cells of interlobular ducts was noted in a few glands (Fig. 1R–T). Only cytoplasmic staining was considered positive for CK15.

CD90, an MSC marker, was highly positive in the interstitial vessels (Fig. 5D). Within the lobules, interstitial CD90-positive cells unrelated to myoepithelial, acinar, or ductal epithelial cells were seen.

### 3.2 Dacryoadenitis

**Fig. 2:**
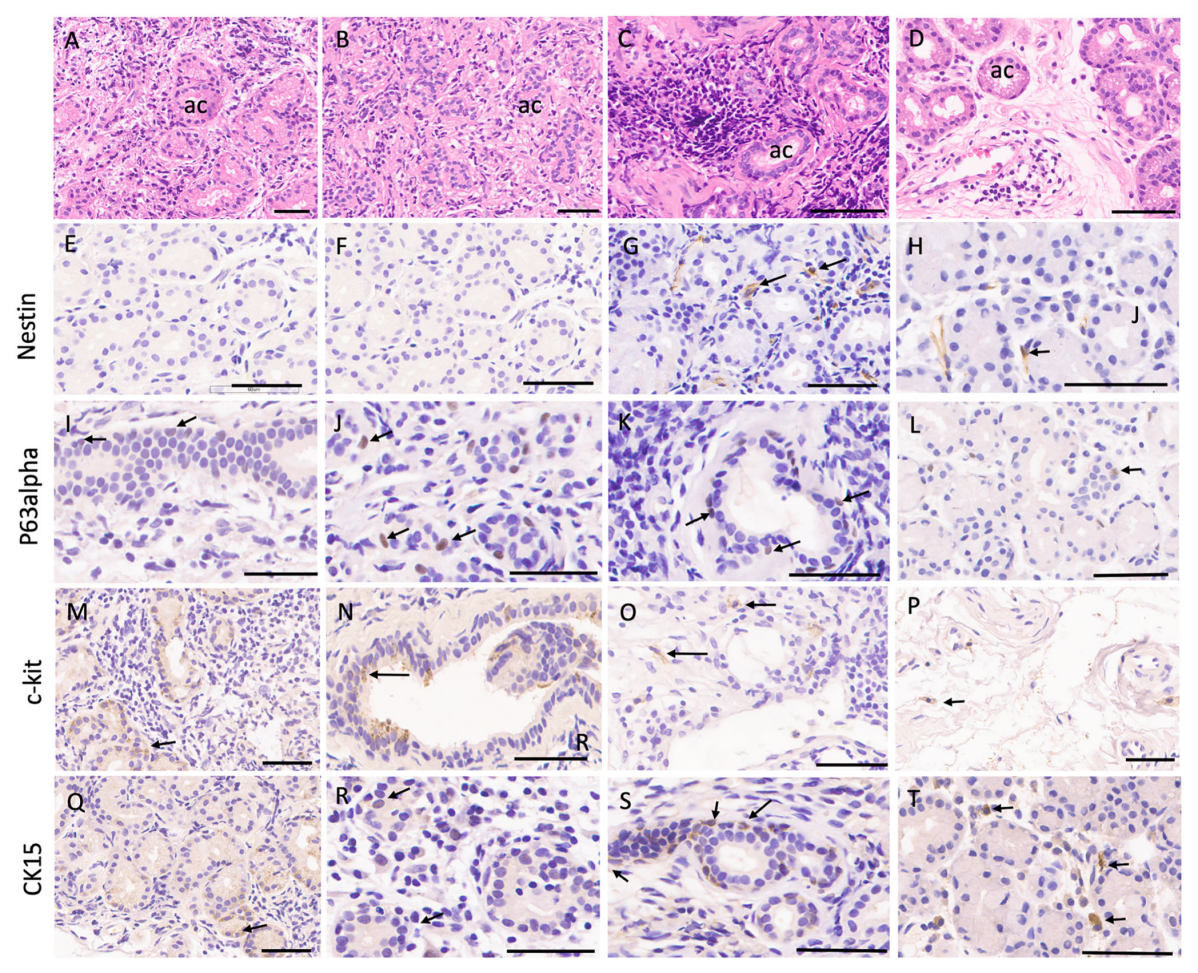
Stem/progenitor cells in human lacrimal glands affected with inflammation (each scale bar represents 60 μm). A-D, Gland biopsy from 34-year-old female (A), 19-year-old male (B), and 50 year-old female (C) dacryoadenitis patient has atrophic acini (ac) interspersed with moderate to severe lymphoplasmacytic infiltrate in three patients (A to C), and mild lymphocytic inflammation in one case (40 year-old male, D). The images in each column represent a sample seen in H&E staining in first row.Nestin expression is faintly positive in periacinar cells in cases 3 &4 (G, H), whereas no expression was seen in E and F.P63alpha expression was noted in a periductal location in all samples (I to L, marked with arrows).Positive c-kit expression in duct-like structures (M, N), and very few scattered stromal cells (O, P).Positive CK15 expression observed in basal cells of ducts (R, S, marked with arrows) in interstitial stromal cells (Q, T).

Of five biopsies, there was a severe diffuse inflammation with increased duct-like structures and acinar atrophy in three samples (Fig. 2; A-C), whereas mild to moderate inflammation with mild acinar atrophy in two samples (Fig. 2; D). The inflammatory cells were pre-dominantly composed of lymphocytes and plasma cells. There was an increase in CD90-positive cells in the inflamed glands, mainly in periductal and interstitial stromal cells (Fig. 5E and F). The c-kit, p63α, CK15, and Nestin-positive cells showed no increase compared to healthy glands (Table 1).

### 3.3 Gene expression analysis

**Fig. 3:**
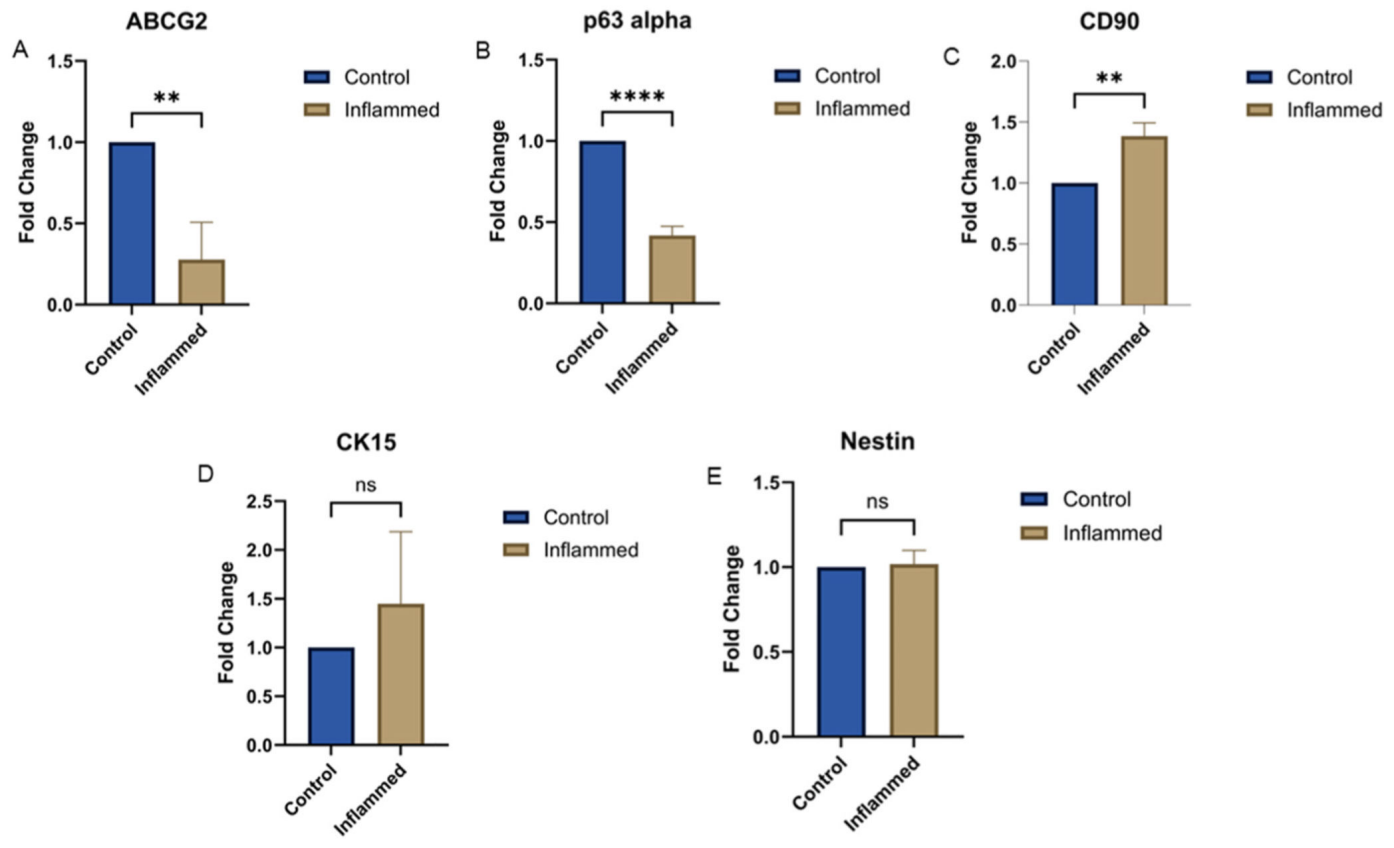
Gene expression studies for stem cell markers in inflamed lacrimal glands The bar graphs represent the relative fold change of inflamed lacrimal gland tissues relative to control with significant downregulation of ABCG2 and p63-α (A–B); considerable upregulation of CD90 (C); non-significant increase in expression of CK-15 (D) with no change in expression of Nestin. Statistical significance is indicated by asterisks (**p < 0.01, ****p < 0.0001).

Gene expression samples had severe dacryoadenitis only. All samples demonstrated detectable expression of CD90, CK15, Nestin, ABCG2, and p63α (Fig. 3A–E). CD90 expression was significantly upregulated in inflamed samples. In contrast, ABCG2 and p63α expression levels were significantly downregulated. CK15 showed a non-significant increase in expression, while Nestin expression remained unchanged compared to control tissues.

### 3.4 Immunofluorescence-based MSC marker expression and quantification

**Fig. 4:**
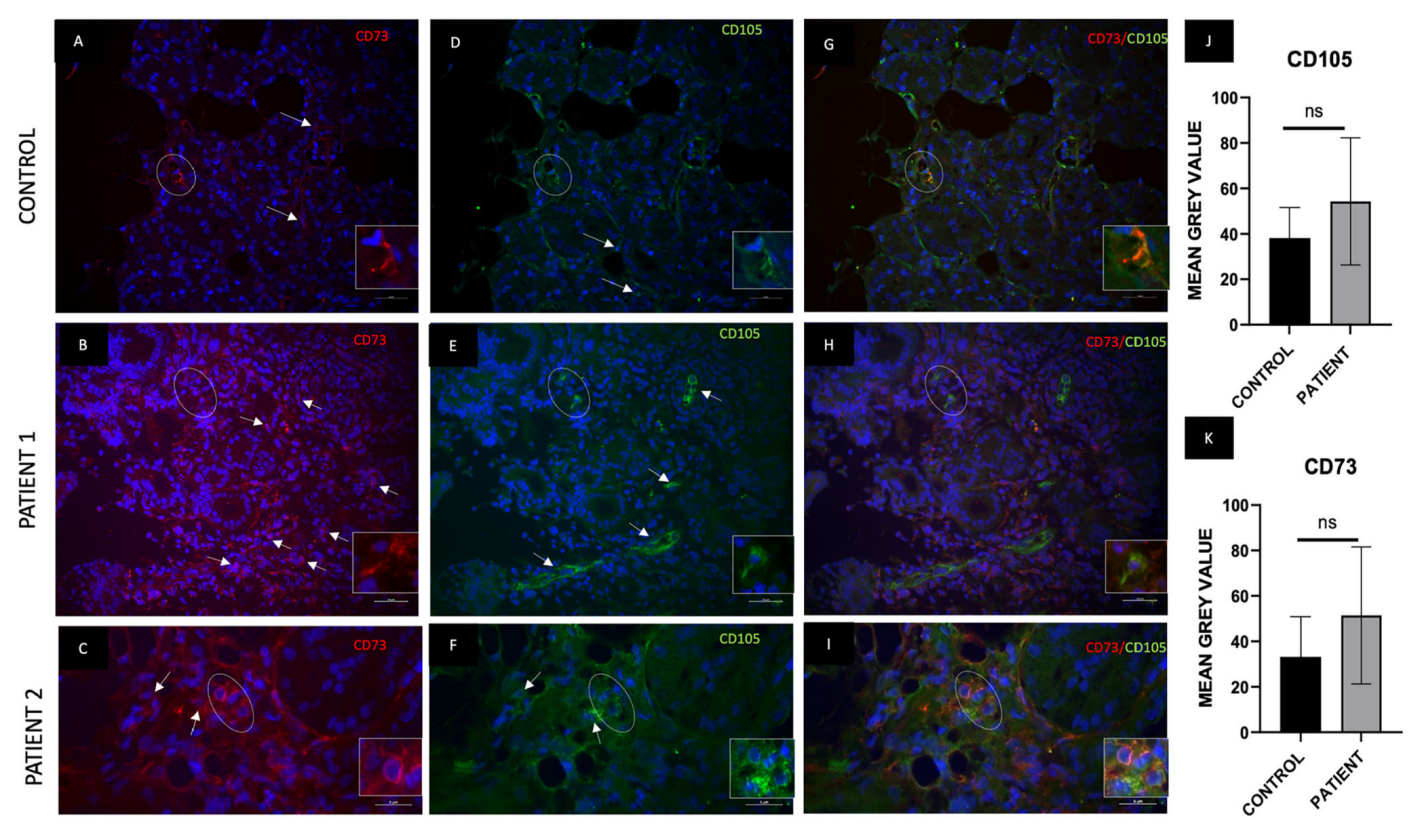
Control and inflamed lacrimal gland tissue samples showed positive expression for stem cell markers **(a**–**c) CD73 and (d**–**f) CD105**. The expression of both markers was restricted mainly to the interstitial region of the glandular acini (shown by white line arrows). (g–i) A few of the cells (indicated by a white dashed circle) stained positive for both CD73 and CD105 in both control and patient tissues (comparatively higher in patients). The w hite inbox shows the double-positive cells at zoom (j–k). The mean fluorescence intensity of CD105 and CD73 showed no significant difference in patients (cumulative data of all patient samples) compared to controls for both markers. (a-i, 40X magnification).

**Fig. 5:**
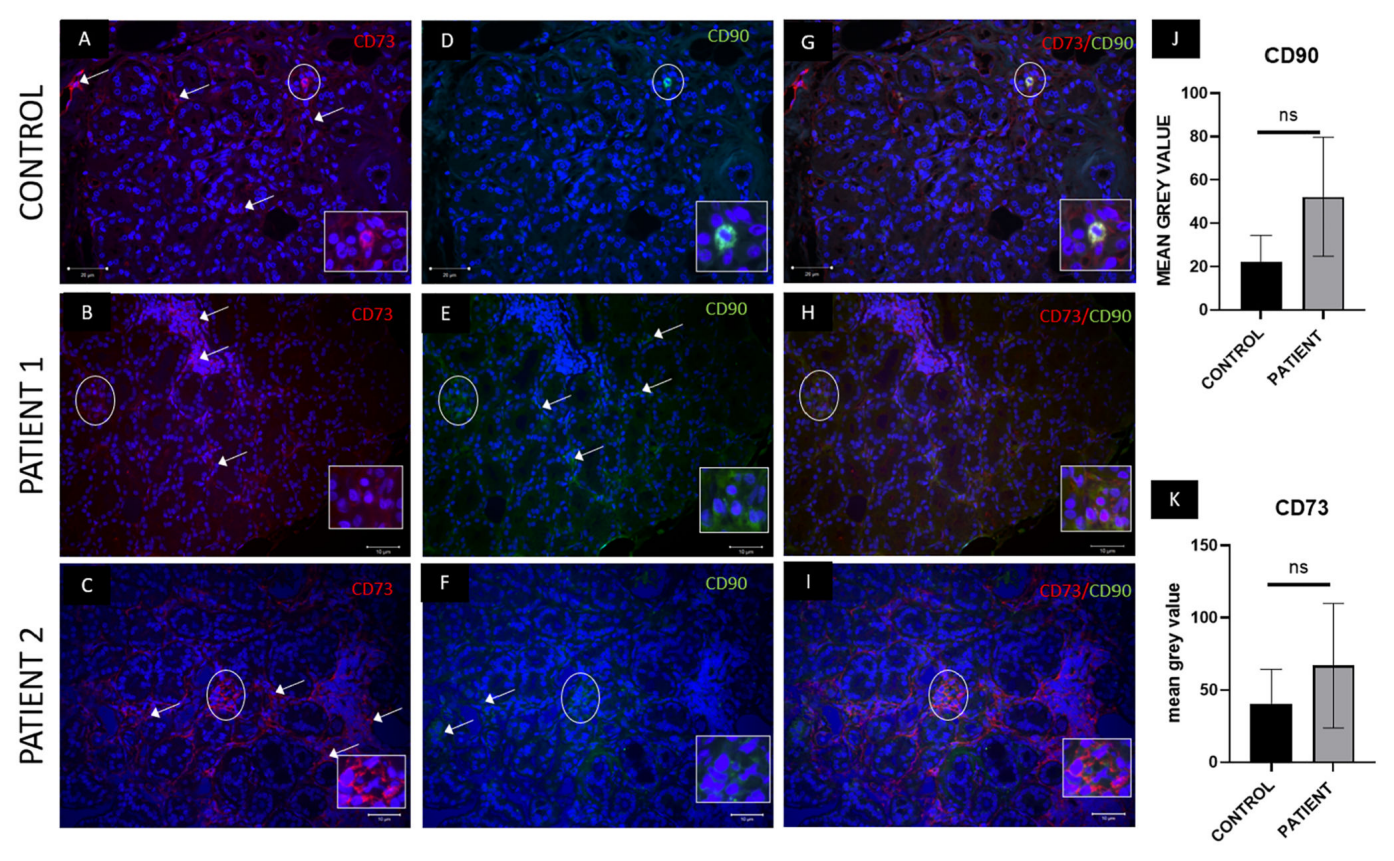
Control and inflamed lacrimal gland tissue samples showed positive for stem cell markers **(a**–**c) CD73 and (d**–**f) CD90**. The expression of both markers was restricted mainly to the interstitial region of the glandular acini, as shown by the white line arrows. (g–i) Few of the cells (indicated by a white dashed circle) stained positive for both CD73 and CD90 in both control and patient tissues (comparatively higher in patients). The white inbox shows the double-positive cells at zoom (j–k). The Mean fluorescence intensity of CD90 and CD73 showed no significant difference in patients compared to controls for both markers. (a-i, 40X magnification).

Double immunofluorescence expression studies were performed to study the location of MSCs in inflamed/healthy lacrimal gland tissues, as CD90 showed an increase in gene expression. The inflamed lacrimal tissues were stained with CD73 & CD105. A positive expression of CD105 (Fig. 4A–C), CD73 (Figs. 4 and 5; D&F), and CD90 (Fig. 5A–C) was observed in both control and inflamed lacrimal glands. However, the expression of CD105 was seen as being majorly restricted to the endothelial cells, while CD73 and CD90 expression was observed in interstitial glandular regions (non-acinar cells) of the lacrimal gland. Further, mean fluorescence intensity for these markers increased in inflamed glands without a significant difference from the control glands (Figs. 4 and 5; J-K). Negligible positive double expression of these markers, i.e., CD105+CD73 (Fig. 4; G to I) and CD90 + 73 (Fig. 5; G to I), was observed in inflamed and control tissues, ruling out demarcation of the CD90-positive cells as stem cells.

## 4 Discussion

The current study demonstrated the progenitor cell expression within normal and inflamed human lacrimal glands. The basal cell layer of interlobular and intercalated ducts and periacinar spindle-shaped cells (likely myoepithelial cells) expressed progenitor markers. Single-cell sequencing and lineage tracing studies of the mice’s lacrimal glands have shown progenitor cells within the basal layer of ducts and intercalated ducts (Basova et al., 2020; Delcroix et al., 2023), which supports our findings as well. The authors also studied whether progenitor cell expression is altered within inflamed glands to restore the atrophied acini. There was a significant increase in CD90 expression on immunostaining and RT-PCR; however, MSC marker expression was positive in the endothelial cells based on double immunostaining with CD73/CD105. No significant increase was noted in c-kit and CK15; p63α and ABCG2 expression were significantly reduced. The gene expression data is from severe dacryoadenitis patients only. A possible reason could be the inhibitory effects of severe inflammation on the activity of progenitor cells. Animal models of lacrimal gland inflammation, the non-obese diabetic mice model of dry eye disease, show a substantial decrease in lacrimal gland progenitor cells and stem cell transcription factors with increased lymphocytic infiltration (Umazume et al., 2015). The interaction between inflammatory cells and stem cells within the lacrimal gland must be studied further.

No lacrimal gland-specific stem cell markers have been reported in the published literature. Development and lineage tracing studies have shown that unipotent lineage-restricted stem/progenitor cells are present in intercalated and interlobular ducts, myoepithelial cells in the adult mouse lacrimal gland (Basova et al., 2020; Farmer et al., 2017). These studies indicate that adult glands have cell-specific progenitor cells for each type of cell population, such as ductal, acinar, and myoepithelial cells. The selected markers in the study represented each of these cell types. Nestin and ABCG2 for acinar progenitor cells, c-kit and CK15 for basal ductal cells, p63α for myoepithelial cells, and CD90, CD73, and CD105 for MSCs. The current study observed different localizations of progenitor markers within the lacrimal gland, such as Nestin and ABCG2 positivity in periacinar cells and CK15 and c-kit positivity in ducts. c-kit + dim/EpCAM+/Sca1-/CD34-/CD45-cells had remarkable progenitor cell properties and salvaged the damaged recipient gland in a mouse model (Gromova et al., 2017). The regenerative capacity of the lacrimal gland has been proposed based on the stemness demonstrated in culture. Normal lacrimal gland biopsies, when cultured, showed cells expressing ABCG2, c-kit, and CD90 (Ackermann et al., 2015; Tiwari et al., 2012). The exact location and expression of progenitor cells in human lacrimal glands have not been studied. The lacrimal gland has pyramidal-shaped epithelial cells arranged in acini surrounded by myoepithelial cells. The intercalated, interlobular, and terminal ducts comprise the ductal tree in the lacrimal gland, similar to salivary glands. The published data (animal studies) on lacrimal gland progenitor cell markers in normal lacrimal gland epithelial cells propose acinar epithelial cells (c-kitdim, EpCAM, Sca1 positive), ductal/myoepithelial cells (Nestin, K14, ABCG2, deltaNp63, K15), and myoepithelial cells (Ki67, alpha-SMA, Nestin) to be the progenitor cells.^19^ Nestin is an intermediate filament that is expressed in dividing cells. In mouse models of lacrimal gland injury, either with interleukin injection or duct ligation, an increase in the stem cell markers Nestin, Ki67, ABCG2, and Sca-1 has been reported (Liu et al., 2017; You et al., 2011; Zoukhri et al., 2008). These Nestin-positive cells were in the interstitium and had an elongated morphology, though it is unclear which cells became Nestin-positive. Some Nestin-positive cells in the mouse lacrimal gland injury model were also positive for alpha SMA and were myoepithelial cells (Zoukhri et al., 2008). In the rat lacrimal gland, myoepithelial cells, other than contracting the acini, were proposed as progenitor cells due to their Nestin, ABCG2, PAX6, SOX2, and Np63 positivity. Earlier studies have proposed MECs or mesenchymal cells as the lacrimal glands’ progenitor cells (Shatos et al., 2012; Makarenkova and Dartt, 2015). The large numbers of MEC progenitor cells seen in the rat lacrimal gland were proposed to be more differentiated than stem cells. The current study found Nestin-positive cells to be periacinar and very similar to the myoepithelial cells in morphology and location. However, their numbers did not increase in inflamed lacrimal glands. Nestin was also expressed in the periductal cells and the cells surrounding the intercalated and interlobular ducts. Nestin-positive cells seem myoepithelial and have progenitor capacity in the human lacrimal gland.

ABCG2 positivity was seen in myoepithelial cells in the normal human lacrimal glands. ABCG2 is a member of the ATP-binding cassette transporters and has been shown in the lateral membranes of acinar epithelial cells. The acinar cells did not show increased ABCG2 expression in dacryoadenitis specimens. Inflammation has likely induced the death of stem/progenitor cells. Cytokeratin expression study of mouse lacrimal gland germ and adult epithelium identified CK15-positive cells in the basal layer of ducts to be present in both stages (Hirayama et al., 2016). The current study also found CK15 expression in the basal cells of interlobular ducts. CK15-positive periductal progenitor cells could be the cells involved in lacrimal gland inflammation repair, as the current study noted an increase (though not statistically significant) in CK15 periductal basal cells in the inflamed glands. During embryonic stages, CK15 is expressed in the lacrimal glands (Hirayama and Hiraya-Ma, 2018). P63 protein is expressed in three isoforms: alpha, beta, and gamma. P63 alpha is implicated in developmental processes and is a marker for progenitor cells in the limbal epithelium. The periductal localization in normal lacrimal glands supports it as a progenitor marker. However, no increase in P63 alpha expression in inflamed glands suggests that these cells differ from CK15-positive basal ductal cells. Single-cell sequencing studies on human lacrimal glands will delineate the progenitor cell population and their specific gene expression. A cluster expressing mitotic genes was identified from single-seq studies of the mouse lacrimal gland Ly6a (Huang et al., 2024). However, further determination of these cell types was not done. They even identified a subset of fibroblasts that expressed stemness genes CD34 and Ly6a (Huang et al., 2024). Further studies are needed to ascertain their role in regeneration.

MSCs are explored as a potential regenerative therapy for lacrimal gland repair. CD90-positive MSCs that were positive on double immunostaining with CD73 and CD105 were located in the stroma of normal human lacrimal glands. Our earlier work has shown the lacrimal gland to contain gland-specific MSCs that differ from bone marrow-derived MSCs (Jaffet et al., 2023). Interstitial MSCs within the lacrimal glands are involved in the repair process (Hawley et al., 2017). Even the secretome of MSCs promoted the growth and secretory capacity of the lacrimal gland epithelial cells (Hawley et al., 2017). However, these were in vitro or animal studies. A randomized clinical trial of intraglandular MSC injection in SS patients showed improvement in the tear volume and ocular staining, similar to the control group receiving DMSO injections (Møller-Hansen et al., 2024). Different possibilities for it not to work could be changes in MSCs due to altered lacrimal gland microenvironment in SS, MSC rejection, or cell migration into the systemic circulation. The lacrimal gland becomes inflamed and atrophic in severe dry eye states like Sjögren’s syndrome. The immunomodulatory effects of MSCs depend on the surrounding inflammatory environment, cytokines, and other factors (Wang et al., 2022). An unfavorable inflammatory environment might impair their activity or induce apoptosis. At low concentrations of IFN gamma and TNF, MSCs instead promote T cell recruitment (Wang et al., 2022). It has also been shown that infused MSCs in GVHD animal models survive for about 3 days and exert their effects through self-initiated apoptosis (Wang et al., 2022). In cases of chronic and low-grade inflammation, MSCs exhibit fewer immunomodulatory effects than during the acute phase. The current study involved dacryoadenitis patients with a mean duration of 5.7 months. Therefore, the findings from acute injury lacrimal gland models do not apply to this disease cohort. In SS patients, bone morphogenetic protein 6 (BMP6) levels are elevated (Xu et al., 2018). In vitro studies have shown that the immunomodulatory effects of MSCs are impaired when exposed to BMP6 (Xu et al., 2018). The interaction of MSCs with B cells also needs to be studied.

Also, no increase in tissue-resident MSCs in inflamed glands, as seen in the current study, might explain the lack of significant change seen with an intraglandular injection of MSCs in SS (Møller-Hansen et al., 2024). The increased MSC gene expression was noted due to inflammatory cells, as double immunostaining localized them inside blood vessels. T cells also express CD73, CD105, and CD90 markers (Deaglio et al., 2007; Gui et al., 1999; Schmidt-Weber et al., 2005). MSCs were not increased in dacryoadenitis specimens in the current study. Also, the localization of MSCs to the periacinar, stromal, or myoepithelial region will help understand their involvement in gland repair mechanisms, which was not the case in inflamed glands.

The limitations of this study include the small number of dacryoadenitis cases and the focus on selective markers. Also, differences in age between normal subjects and patients should be considered. Gene expression data comes from severe cases with significant acinar atrophy, so understanding changes at various inflammation levels is important for future research. Strengths include the immunohistochemical localization of different stem/progenitor cell markers in the normal human lacrimal gland and, for the first time, in inflamed human lacrimal glands to evaluate progenitor marker expression. It remains to be explored whether MSCs can restore atrophied lacrimal glands with inflammation, as seen in severe dry eyes caused by SS.

The normal human lacrimal gland has progenitor cells around the intercalated and interlobular ducts. In inflamed glands, a mild increase in the expression of periductal progenitor cells occurs, but there is no increase in periacinar progenitor marker or MSC expression. The glandular inflammation seemed to suppress the progenitor cells. Studying lacrimal gland behavior in inflammation states, like chronic non-specific dacryoadenitis, would help us understand gland biology and the interactions of stem cells and immune cells.

## Figures and Tables

**Table 1:** Progenitor cell expression in normal human lacrimal gland and gland biopsies of dacryoadenitis.

Progenitor cell marker	Location & intensity (in nine normal glands)
c-kit	Stromal 1+ (9/9)
Nestin	Periacinar- 1+ (1/9), 2+ (5/9), 3+ (3/9)
	Periductal- 1+ (7/9), 2+ (2/9)
CK15	Periductal- 1+ (2/9), 2+ (2/9), 3+ (5/9)
	Stromal- 2+ (5/9), 3+ (4/9)
ABCG2	Periacinar- 1+ (7/9), 2+ (2/9)
P63α	Periductal- 1+ (1/9), 2+ (7/9), 3+ (1/9)
CD90	Stromal 1+ (7/9)

**Table 2:** Qualitative expression analysis of progenitor cells in normal human lacrimal glands.

Progenitor cell marker (% positive cells, Mean ± SD)	Normal (n = 9)	Dacryoadenitis (n = 5)	P value
c-kit	0.006 ± 0.001	0.01 ± 0.007	0.06
Nestin	0.73 ± 0.06 (periacinar) 0.47 ± 0.03 (periductal)	0.22 ± 0.02	0.38
CK15	0.35 ± 0.09	0.46 ± 0.05	0.57
ABCG2	0.46 ± 0.1	0.07 ± 0.004	0.9
P63a	0.04 ± 0.03	0.12 ± 0.03	0.06

**Table 3:** List of primers used for quantitative real-time PCR (Fwd- Forward primer, Rev-Reverse primer, Product size).

Gene	Primer sequence	Product Size (bp)
**Beta actin**	**Fwd** TCTACAATGAGCTGCGTGTG **Rev** GGTGAGGATCTTCATGAGGT	314
**Nestin**	**Fwd** CAACGTACACCCCGATCCTG **Rev** GGATCTCCCCAGAACCCAAC	147
**CD90**	**Fwd** AGCATCGCTCTCCTGCTAAC **Rev** CTGGTGAAGTTGGTTCGGGA	230
**ABCG2**	**Fwd** GGGTTCTCTTCTTCCTGACGACC **Rev** TGGTTGTGAGATTGACCAACAGACC	399
**p63α**	**Fwd** ACCTGGAAAACAATGCCCAGA **Rev** GAGGTGGGGTCATCACCTTG	369
**CK15**	**Fwd** TGGAGATCGACAATGCCAGG **Rev** GTCAGCTCATCCAGGACTCG	124

## Data Availability

No data was used for the research described in the article.
